# Does it matter whether the recipient of patient questionnaires in general practice is the general practitioner or an independent researcher? The REPLY randomised trial

**DOI:** 10.1186/1471-2288-8-42

**Published:** 2008-06-27

**Authors:** James A Desborough, Peter Butters, Debi Bhattacharya, Richard C Holland, David J Wright

**Affiliations:** 1 School of Chemical Sciences and Pharmacy, University of East Anglia, Norwich, UK; 2 Bungay Medical Practice, Suffolk, UK; 3 School of Medicine, Health Policy and Practice, University of East Anglia, Norwich, UK

## Abstract

**Background:**

Self-administered questionnaires are becoming increasingly common in general practice. Much research has explored methods to increase response rates but comparatively few studies have explored the effect of questionnaire administration on reported answers.

**Methods:**

The aim of this study was to determine the effect on responses of returning patient questionnaires to the respondents' medical practice or an independent researcher to questions relating to adherence and satisfaction with a GP consultation. One medical practice in Waveney primary care trust, Suffolk, England participated in this randomised trial. Patients over 18 years initiated on a new long-term medication during a consultation with a GP were randomly allocated to return a survey from their medical practice to either their medical practice or an independent researcher. The main outcome measures were self reported adherence, satisfaction with information about the newly prescribed medicine, the consultation and involvement in discussions.

**Results:**

274 (47%) patients responded to the questionnaire (45% medical practice, 48% independent researcher (95% CI -5 to 11%, p = 0.46)) and the groups appeared demographically comparable, although the high level of non-response limits the ability to assess this. There were no significant differences between the groups with respect to total adherence or any of the satisfaction scales. Five (4%) patients reported altering doses of medication in the medical practice group compared with 18 (13%) in the researcher group (P = 0.009, Fisher's exact test). More patients in the medical practice group reported difficulties using their medication compared to the researcher group (46 (35%) *v *30 (21%); p = 0.015, Fisher's exact test).

**Conclusion:**

Postal satisfaction questionnaires do not appear to be affected by whether they are returned to the patient's own medical practice or an independent researcher. However, returning postal questionnaires relating to detailed patient behaviours may be subject to response biases and further work is needed to explore this phenomena.

## Background

Patient surveys are becoming an increasingly common tool to measure quality and improve services [1]. Although doubts remain over the validity of these surveys in assessing quality of care [2,3] the current General Medical Services (GMS) contract provides additional financial remuneration for conducting patient surveys. Furthermore, it is proposed that in the future these surveys will require patients to provide even greater detail about their consultations [4].

While much research has investigated the administration of patient surveys, the majority has explored methods to increase response rates [5]. Intense survey methods have been reported to yield greater response rates [6], however postal questionnaires appear to remain the most cost effective mode of administration [7,8]. It has also been suggested that responses to postal questionnaires are likely to be more accurate than other methods when enquiring about sensitive issues such as health and behaviour [9-11].

It is unsurprising therefore that self-administered postal questionnaires have been widely adopted as the survey method of choice. However, it is not known how subtle differences within this method of administration affects elicited responses [8,10]. Results from a small medical practice audit of patient self reported adherence suggested no patients deviated from the instruction given to them by their doctor when the self administered questionnaire was returned to the patients own medical practice. This is in sharp contrast to literature where self reported adherence to medication for chronic conditions is usually 50%, and therefore led us to the hypothesis that patients returning questionnaires regarding potentially "deviant" behaviour (in this case non-adherence to medication) directly to their medical practice might be unwilling to report this.

Questions on adherence may be subject to additional response biases if administered by the patient's own medical practice. Fear of reprisals and the reluctance to express aversion to medicines may increase bias of previously validated questionnaires despite assurances of confidentiality [12-14]. In addition, questionnaires which include questions regarding satisfaction with the doctors consultation and the information provided may also demonstrate socially desirable responses with higher levels of reported satisfaction if returned directly back to the medical practice of the patient. Although a number of researchers have required patients to submit responses to independent researchers [15,16], greater understanding of this administrative method is needed.

We therefore performed a randomised controlled trial with the primary aim of determining whether patients returning questionnaires relating to their GP consultation to either their medical practice or an independent researcher affects patients' reports of their adherence. The secondary aims of this research to explore the components of GP consultations which affect medication adherence will be reported later.

## Methods

### Sample

The study was conducted in one medical practice in Suffolk and approved by the Norfolk research ethics committee prior to commencement of data collection. During the study period, the practice had a list size of 10,000 patients, with 10 GPs conducting consultations. Patients over 18 years, who had been initiated on to a long-term medication by the GP in a consultation at the practice, were invited to participate. The sample only included patients prescribed one de novo solid oral dosage form (or inhaler) medication with a specified daily dosage (excluding PRN medication) and did not included courses of medicines prescribed for the treatment of acute conditions. Patients were randomised to return their questionnaire to either the medical practice or an independent researcher by the opening of randomly allocated opaque envelopes in chronological order, blocked in varying lengths and stratified by GP.

### Procedures

The study was performed between November 2005 and August 2006, as a randomised control trial. Potential participants were not aware of the randomisation and were only told the research was interested in their recent visit to the doctor. Those allocated to the medical practice group were sent a questionnaire with an introductory paragraph from the medical practice and a pre-paid reply envelope addressed to the medical practice. Those allocated to the researcher group were sent a questionnaire with an introductory paragraph from the researcher and a pre-paid reply envelope addressed to the researcher (see additional files 1 and 2). The questionnaires were otherwise identical and sent to patients with the consent form seven days after the index consultation. The allocation was performed by a receptionist and both GPs and patients were blinded to the allocation. Non-responders were sent a second posting of the questionnaire after two weeks.

### Measurements

The questionnaire was developed from existing tools available from the medicine partnership [17] designed to explore components important for achieving concordance. The Medication Adherence Report Scale (MARS)[18] was used to measure adherence to the newly prescribed medication. This scale requires patients to report their frequency of behaviour relating to five non-adherence behaviours; forgetting, missing, altering, taking less and stopping medication where 5 = never, 4 = rarely, 3 = sometimes, 2 = often and 1 = always. The Satisfaction with Information about Medicines Scale (SIMS)[19] is a validated questionnaire providing a profile of patients' satisfaction with the information they have received about their newly initiated medication. It consists of 17 items requesting patients to rate the amount of information received on each item as: 'too much', 'about right', 'too little', 'none received' and 'none needed'. The Perception of Involvement in Discussions (PID) [20] requires patients to rate how much they agree with four statements relating to their involvement in discussions on a 5-point Likert scale ranging from 'strongly agree' to 'strongly disagree'. The Medication Interview Satisfaction Scale (MISS-21)[21] asks patients to rate 21 statements about their satisfaction with their consultation on a 7-point Likert scale ranging from 'very strongly disagree' to 'very strongly agree'. The questionnaire additionally included a checklist of potential difficulties patients may have with using their medication, e.g. opening lids or swallowing tablets. Patient characteristics were extracted from practice medical records by a researcher on patient completion of the questionnaire. These included: gender, age, living status, number of prescribed daily medications, number of prescribed 'when required' medication, current active problems and details of the newly prescribed medications.

### Sample size calculation

Initial responses to a practice audit of adherence (a survey returned directly to the medical practice) demonstrated 100% of patients scoring 25 (perfect score) using the MARS questionnaire. A previous study using the MARS questionnaire returned to a researcher, reported 80% of patients scoring 25 [22]. Therefore it was calculated that 274 patients (137 in each group) would be required to detect a conservative 95 versus 85% difference in patients scoring 25 using the MARS questionnaire with 80% power and using a 5% significance level. There was a paucity of data on the likely response rates to inform calculations of how many patients would need to be sent questionnaires to yield an achieved sample size of 274. In the absence of such data, identification and recruitment of study participants continued until 274 completed questionnaires had been received.

### Data analysis

Responders and non responders were compared with respect to age and gender. The medical practice and researcher groups were compared according to patient characteristics and the newly prescribed medicine. MARS scores were dichotomised into either 'adherent' (score = 25) or 'partially adherent' (score < 25). This was further subdivided by dichotomising each of the non-adherent behaviours as not occurring (score = 5) or occurring (score < 5). The proportion of patients in each group who reported adherence was compared using the Fisher's exact test with confidence intervals calculated assuming the normal approximation to the binomial. The SIMS, MISS-21 total scores and subscale scores and response to each individual "Perception of involvement in discussions" questions were compared between groups using the Mann Whitney U test as distributions were expected to be skewed. The proportion of patients reporting difficulties with medication in each group was compared using the Fisher's exact test. Data manipulation was carried out using SPSS version 14 with statistical significance set at 5%.

## Results

**Figure 1:**
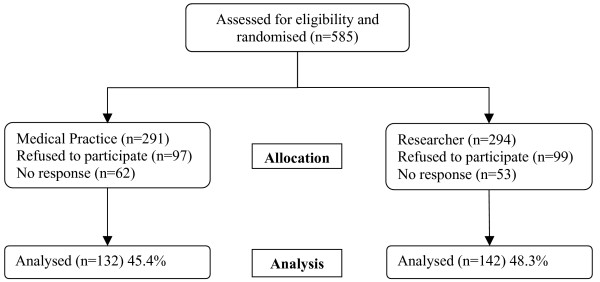
Flowchart showing progress of patients through trial.

Invitations to participate were extended to 585 patients; 274 (46.8%) patients agreed and completed the questionnaire (figure 1). There was no difference in the recruitment of patients between each randomisation group (45% medical practice *vs*. 48% researcher, (95% CI -5 to 11%) p = 0.508), however non-responders were younger than responders (mean age = 59.2 *v *63.4 years, p = 0.011) but response was not related to gender. The remainder of this paper focuses on the responders and table 1 shows both groups were very similar at baseline. However, with less than a 50% response rate, the study groups may not be comparable with respect to any unobserved variables.

**Table 1:** Baseline comparison of medical practice and researcher group patients. Values are numbers (percentages) unless stated otherwise.

Characteristic	Medical Practice (n = 132)	Researcher (n = 142)
Female	82 (62.1)	88 (62.0)
Median (Q_1_–Q_3_) Age (years)	62.0 (51.3–74.8)	65.0 (54.0–74.0)
Living alone	23 (17.4)	25 (17.6)
Median (Q_1_–Q_3_) Daily drugs	3.0 (2.0–5.8)	3.0 (2.0–5.0)
Median (Q_1_–Q_3_) PRN drugs	1.0 (0.0–1.0)	1.0 (0.0–2.0)
**Active problems**		
Diseases of circulatory system	89 (67.4)	92 (64.8)
Hypertension	51 (38.6)	55 (38.7)
Ischemic Heart Disease	18 (13.6)	13 (9.2)
Cerebrovascular Disease	13 (9.8)	12 (8.5)
Other forms of heart disease	7 (5.3)	12 (8.5)
Asthma/COPD	29 (21.9)	35 (24.6)
Diabetes Mellitus	23 (17.4)	23 (16.2)
Disorders of the thyroid gland	14 (10.6)	12 (8.5)
Mood [affective] disorders	16 (12.1)	21 (14.8)
Eye & adnexa	12 (9.1)	14 (9.9)
Digestive system	25 (18.9)	29 (20.4)
Skin & subcutaneous tissue	11 (8.3)	10 (7.0)
Musculatory system & connective tissue	34 (25.8)	44 (30.9)
Genitourinary	24 (18.2)	23 (16.2)
Other	34 (25.8)	23 (16.2)
**Newly prescribed medication**		
Cardiovascular system	66 (50.0)	70 (49.3)
Diuretics	3 (2.3)	12 (8.5)
Beta-blockers	5 (3.8)	9 (6.3)
ACE inhibitors or ARAs	21 (15.9)	12 (8.5)
Calcium-channel blockers	3 (2.3)	14 (9.9)
Lipid regulating drugs	19 (14.4)	12 (8.5)
Respiratory system	14 (10.6)	10 (7.0)
Central nervous system	12 (9.1)	12 (8.5)
Endocrine system	18 (13.6)	21 (14.8)
Obstetrics & gynaecology	13 (9.8)	14 (9.9)
Others	9 (6.8)	15 (10.6)
**Dosage form**		
Tablets or Capsules	114 (86.4)	132 (93.0)
Inhalers	13 (9.8)	8 (5.6)
Others	5 (3.8)	2 (1.4)
**Dosage frequency**		
Less than daily	4 (3.0)	6 (4.2)
Daily	103 (78.0)	118 (83.1)
More than daily	24 (18.2)	17 (12.0)
Unknown	1 (0.8)	1 (0.7)

(Q_1_–Q_3_) = lower quartile – upper quartile

ACE = Angiotensin-converting enzyme inhibitors

ARAs = Angiotensin-II receptor antagonists

### Adherence

**Figure 2:**
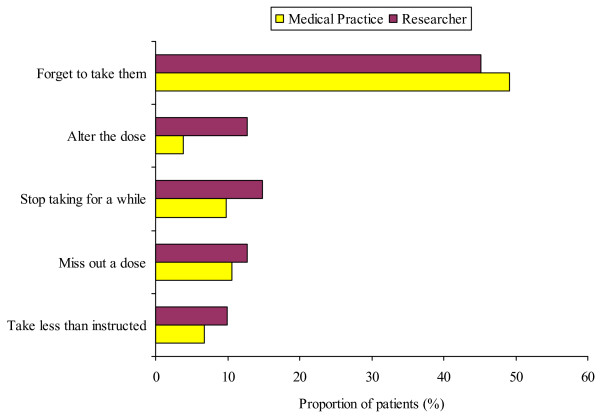
The proportion of patients reporting the different types of non-adherent behaviours of the medication adherence report scale.

The overall proportion of patients reporting being adherent was markedly lower than expected from the previously reported rate of 100% in the medical practice audit or 80% reported by Bhattacharya [22]. There was no significant difference in the proportion of patients who reported being adherent between the medical practice and researcher groups (40.9% *v *45.1%, p = 0.54 difference in proportions 95% CI -14.7% to 23.1%). Figure 2 shows the exploratory secondary analysis of the types of non-adherent behaviour. This figure reveals that a smaller proportion of patients reported altering doses in the medical practice group (3.8% *v *12.7%, p = 0.009, difference in proportions 95% CI 2.5% to 15.3%). There were no differences reported in the frequency of the other types of non-adherent behaviour or the proportion of missing survey items between the groups.

### Satisfaction

Table 2 shows the total and subscale scores for the SIMS and MISS-21 survey sections. There was no difference in responses between groups; however, only 32.5% of the medical practice group and 29.1% of the researcher group were completely satisfied with the amount of information they had received about their newly prescribed medicine. In response to individual questions in this section of the questionnaire, only eight patients reported receiving too much information from their GP. MISS-21 scores were similar; 35.6% and 31.0% of patients reporting satisfaction with all items respectively.

**Table 2:** Satisfaction scales scores

Satisfaction Scale	Possible Score Range	Medical Practice (N = 132)			Researcher (N = 142)		
		
		Score Mean (SD)	Satisfied* N (%)	Completed items** N (%)	Score Mean (SD)	Satisfied* N (%)	Completed items** N (%)
**SIMS**							
Action and usage	0–9	7.5 (1.8)	54 (40.9)	119 (90.2)	7.5 (1.9)	60 (42.3)	132 (92.9)
Potential problems of medication	0–8	6.2 (2.1)	50 (37.9)	117 (88.6)	6.3 (2.1)	55 (38.7)	130 (91.5)
**Overall satisfaction**	**0–17**	**13.8 (3.4)**	**37 (28.0)**	**114 (86.4)**	**13.8 (3.6)**	**37 (26.1)**	**127 (89.4)**
**MISS-21**							
Distress relief	6–42	30.7 (4.9)	63 (47.7)	121 (91.7)	30.5 (5.0)	56 (39.4)	131 (92.3)
Communic-ation comfort	4–28	21.4 (3.7)	86 (65.2)	123 (93.2)	21.6 (4.1)	88 (62.0)	132 (93.0)
Rapport	8–56	45.0 (7.4)	92 (69.7)	124 (93.9)	44.8 (6.4)	104 (73.2)	135 (95.1)
Compliance intent	3–21	16.0 (3.0)	93 (70.5)	126 (95.5)	16.4 (2.9)	108 (76.1)	137 (96.5)
**Overall satisfaction**	**21–147**	**112.8 (15.4)**	**47 (35.6)**	**113 (85.6)**	**113.2 (15.1)**	**44 (31.0)**	**124 (87.3)**

*Participants who marked 'about right' or 'none needed' on SIMS scale items or who scored ≥ 5 on MISS-21 scale items

**Participants who completed all items in the scale

### Involvement in discussions

There were no significant differences between the groups in reporting involvement in discussion about treatment (table 3). In both groups, approximately half the respondents agreed that they received enough information and were given responsibility for deciding how to deal with their health problem. Yet only 13% of respondents reported being asked to choose a treatment for their health problem.

**Table 3:** Perception of involvement in discussions. Values are numbers (percentages) unless otherwise stated.

	Medical Practice (N = 132)		Researcher (N = 142)	
	
	Agree with item	Completed*	Agree with item	Completed*
The doctor gave me responsibility for deciding how to deal with my health problems	60 (45.5)	124 (93.9)	50 (35.2)	134 (94.4)
The doctor asked me to choose a treatment for my health problem	18 (13.6)	122 (92.4)	18 (12.7)	132 (93.0)
The doctor gave me enough information to make my own decision about treatment	65 (49.2)	127 (96.2)	69 (48.6)	137 (96.5)
The doctor did not ask my opinion about my medicines	34 (25.8)	123 (93.2)	42 (29.6)	135 (95.1)

*Patients who completed the item

### Difficulties using medication

Patients in the medical practice group were significantly more likely to report difficulties using their medication (34.8% medical practice *v *21.1% researcher, p = 0.015). The most frequently reported difficulties were opening lids and remembering times of day and days of the week (Table 4).

**Table 4:** Potential difficulties using medication. Values are numbers (percentages) unless stated otherwise

**Difficulty**	**Medical Practice (n = 132)**	**Researcher (n = 142)**
Opening lids	16 (12.1)	12 (8.5)
Using blister packs	9 (6.8)	9 (6.3)
Understanding directions	3 (2.3)	2 (1.4)
Swallowing tablets	8 (6.1)	5 (3.5)
Splitting tablets	3 (2.3)	4 (2.8)
Pouring liquids	1 (0.8)	2 (1.4)
Reading labels	0 (0.0)	4 (2.8)
Managing eye/ear drops	6 (4.5)	4 (2.8)
Picking up tablets	6 (4.5)	5 (3.5)
Using other devices	1 (0.8)	1 (0.7)
Remembering days/times	12 (9.1)	4 (2.8)
**Overall**		
**Any difficulties reported**^1^	46 (34.8)*	30 (21.1)*

^1 ^Patients who reported one or more difficulties using their medication. *p < 0.05 Fisher's exact

## Discussion

This is the first randomised controlled study to investigate whether patients give different responses to questionnaires regarding detailed aspects of their consultation including medication adherence which they return to their own medical practice, in comparison to those returned to an independent researcher. The survey was conducted from one medical practice in Suffolk.

### Summary of main findings

The study found no significant differences in the proportion of patients reporting being adherent or in the satisfaction levels of components of the consultation between submitting responses to the medical practice or an independent researcher. Significant differences did exist in the secondary outcomes of reporting reasons for non-adherence and difficulties using medication. Fewer patients reported altering doses of medication and more patients reported difficulties using their medication in the medical practice group.

### Comparison with existing literature

Consistent with previous data [23,24], we found approximately 50% of patients reported non-adherence when classifying patients as non-adherent if they scored < 25 on MARS. However, the mean MARS scores were higher than previously reported [25] suggesting patients were either: only reporting one type of non-adherent behaviour, or more likely, patients had restricted opportunity to deviate because of the limited time between initial prescribing and survey completion. MARS and other self reported adherence measures tend to overestimate adherence, but are highly accurate for patients who report non-adherence (minimal false positives) [26,27]. This study may have also over estimated the absolute level of non-adherence. With a response rate of less than 50% only those more adherent participants may have responded, although we believe that this effect would be consistent in both groups. All satisfaction scale scores were similar to previous studies [20,21,26].

Comparisons of self administered questionnaire techniques (email, postal, handing out questionnaires to patients) appear to find no differences in responses for health satisfaction surveys [28,29] and this study suggests administration differences within the postal technique will also not affect reported satisfaction. Despite equal assurances of confidentiality, we believe the differences observed between the two groups in terms of reporting adherence and difficulties using medication may be a result of patients not wanting to appear responsible for their non-adherence. Though it is possible that these secondary outcomes are non significant and should only be considered as exploratory. Earlier studies have described how patients find it difficult to express an aversion to medicines directly to their GP [12,13], thus reporting forgetting rather than altering doses may be more socially desirable for surveys returned directly to the patient's medical practice. The increase in reported problems using medicines in the medical practice group also corresponds to socially desirable responses, as patients attempt to justify their non-adherence as unintentional and not a conscious deviation from instructions.

There was no difference in response rate to the overall questionnaire or individual items on the questionnaire, which contrasts previous research comparing response rates between similar groups [30]. Smith *et al*.'s study used a general health survey on heart disease to compare response rates between questionnaires introduced and returned to the patients' general practitioner and those introduced and returned to a doctor at a research unit. The overall response rate in the general practitioner group was 85% compared to 75% in the research unit arm. There are a number of possible explanations why this study received a higher response rate overall and a difference in response between the two groups not observed in our study. Smith *et al.'s *study occurred more than 20 years ago and since this time there has been a general decline in response rates to questionnaires. Furthermore it only targeted patients aged between 40 and 59 years with non response higher in younger age groups [31]. The content of the questionnaire (a general health survey) may explain the difference in response between the two groups, as the perceived importance of the questionnaire in the medical practice arm may have been increased, an effect not demonstrated in our questionnaire regarding a specific consultation.

### Strengths and limitations of this study

The results of this study should be viewed in the context of several limitations. This study occurred in one medical practice with a practice size of 10,000; although all 10 GPs were involved, it may not be generalisable to other practices, especially very small single handed practices where patients consult the same GP. The results therefore should be replicated in a wider variety of settings. However, the poor response rate (47%) makes it impossible to be sure whether randomisation ensured comparability between the groups at baseline, though for the recorded baseline variables the two groups were comparable. The response rate and age difference of non-responders was consistent with other similar postal questionnaires [32,33]. However it may be necessary to explore other more intensive modes of administration to see if responses to questions requesting similar information are significantly different. Twenty statistical tests were performed on secondary outcomes in this study, as it was not powered for these analyses one false positive result would be expected and we made no correction for multiple testing. Therefore our significant findings should be considered exploratory. The patients' ability to identify the survey recipient may have been decreased by ethical requirements to enclose a covering letter from the medical practice. This letter was on practice headed paper and asked patients to read the enclosed information regarding a research project. To determine the true extent of response differences the ideal research design would have been a factorial design involving four cells (sent by practice returned to practice; sent by practice returned to independent researcher; sent by independent researcher returned to practice; sent by independent researcher returned to independent researcher). However, this design is not possible with current ethical and data protection concerns, hence the pragmatic nature of our design. Additional qualitative analysis would have helped to determine the significance of the covering letter and explore reasons for specific responses. As a randomised controlled trial it was hoped that this study would be able to detect genuine differences in responses between respondents that were not a result of different settings which is a general limitation of much of the literature on modes of questionnaire administration [10]. However, the level of non-response makes it impossible to assess if the groups were comparable at baseline.

## Conclusion

This study found no difference in recorded adherence or satisfaction between our two groups. Assuming the groups were comparable at baseline it therefore supports the validity of returning existing patient satisfaction questionnaires used in the current GMS contract to the patients' medical practice. Our secondary findings led us to the hypothesis that if questionnaires are extended to explore more detailed experiences of consultations and patient behaviours, responses may be subject to additional response bias. In our case we believe social desirability bias to be responsible for the differences observed and any method able to reduce this phenomena with behavioural questions is of benefit to future questionnaires [34].

In the future research should compare questionnaire response to measurable outcomes where possible to help determine the accuracy of responses. While maximising response rates remains important, greater consideration should be placed on the quality of responses for questionnaires which explore patient behaviours. Every aspect of survey administration should be carefully considered as it may confer important affects on responses.

## Competing interest statement

The authors declare that they have no competing interests.

## Authors' contributions

All authors contributed to the concept and design of the study. PB was involved in data collection. DJW, RCH and DB contributed to analysis. JAD was responsible for data collection, analysis and writing this manuscript. All authors read, commented on and approved the manuscript.

## Pre-publication history

The pre-publication history for this paper can be accessed here:



## Supplementary Material

Additional file 1 **GP questionnaire version 3**. Copy of the questionnaire with an introductory paragraph from the medical practice Click here for file

Additional file 2 **Researcher questionnaire version 3**. Copy of the questionnaire with an introductory paragraph from the researcher Click here for file
