# Supplementary material for: Does it matter whether the recipient of patient questionnaires in general practice is the general practitioner or an independent researcher? The REPLY randomised trial
Source: BMC Med Res Methodol. 2008 Jun 27;8:42. doi: 10.1186/1471-2288-8-42 (PMC2447849; doi:10.1186/1471-2288-8-42)
Supplement: Additional file 2 — Researcher questionnaire version 3. Copy of the questionnaire with an introductory paragraph from the researcher [file 1471-2288-8-42-S2.pdf]

Study Number: ______

How you view your care

I am a researcher at the University of East Anglia and would appreciate your views about your recent visit to the doctor and the medicines the doctor prescribed for you.

The questionnaire should take no longer than 10 minutes to complete. Your responses to this questionnaire will remain confidential. Your honesty would be greatly appreciated. On completion, please return the questionnaire in the stamped, addressed envelope provided to the University of East Anglia. If you do not wish to complete this questionnaire, please return the uncompleted questionnaire in the stamped, addressed envelope provided.

Section 1 Questions about using your medicines

- Many people find a way of using their medicines which suits them.
- This may differ from the instructions on the label or from what their doctor has said.

Here are some ways in which people have said that they use their medicines

For each statement, please tick the box that best applies to you for **your newly prescribed medicine(s)**

	Your own way of using your medicines	Always	Often	Sometimes	Rarely	Never
1	I forget to take them					
2	I alter the dose					
3	I stop taking them for a while					
4	I decide to miss out a dose					
5	I take less than instructed					

For each statement, please tick the box that best applies to you for all of your **other medicines prescribed for regular use.**

If you are prescribed no other medication for regular use, please go to section 2

	Your own way of using your medicines	Always	Often	Sometimes	Rarely	Never
6	I forget to take them					
7	I alter the dose					
8	I stop taking them for a while					
9	I decide to miss out a dose					
10	I take less than instructed					

Section 2 Information about your medicines

- Please rate the information you have received from your doctor about each of the following aspects of your newly prescribed medicine.

	Have you received enough information about:	Amount of information received Too Much	About Right	Too Little	None Received	None needed
11	How to take your medicine					
12	What your medicine is called					
13	What your medicine is for					
14	What it does					
15	How it works					
16	How long it will take to act					
17	How you can tell if it is working					
18	How long you will need to be on the medicine					
19	Whether the medicine has any unwanted effects (side-effects)					
20	What are the risks of you getting side-effects					
21	What you should do if you experience unwanted effects (side-effects)					
22	Whether you can drink alcohol while you are on this medicine					
23	Whether the medicine will interfere with other medicines					
24	Whether the medicine will affect you sex life					
25	Whether the medicine will make you feel drowsy					
26	What you should do if you forget to take a dose					
27	How to get a further supply					

Section 3 Problems using your medicines

28 Do you have difficulty taking your medicines due to any of the following reasons?

Opening lids	❑	Using blister packs	❑	Understanding the directions	❑
Swallowing tablets	❑	Splitting tablets	❑	Pouring liquid medicine	❑
Injecting insulin	❑	Reading labels	❑	Managing eye/ear drops	❑
Picking up tablets	❑	Other devices	❑	Remembering time of day/week	❑

Other ❑ Please specify: ____________________

Section 4 Your involvement in discussions about treatment

- We want to find out about how you feel about discussions you have had with your doctor about your treatment.
- Please read each statement below and then tick one of the boxes

How much do you agree with the following statements?

(Please tick only one box for each question)

		Strongly agree	Agree	Neutral	Disagree	Strongly disagree
**29**	The doctor gave me responsibility for deciding how to deal with my health problem					
**30**	The doctor asked me to choose a treatment for my health problem					
**31**	The doctor gave me enough information to make my own decision about treatment					
**32**	The doctor did not ask my opinion about my medicines					

Section 5 Satisfaction with your medical consultation

- Please think about the consultation you have recently had with your doctor and indicate whether you agree or disagree with each of the following statements

Tick the box, to show how much you agree or disagree with each statement

		Very strongly disagree	Strongly disagree	Disagree	Uncertain	Agree	Strongly agree	Very strongly agree
33	The doctor told me just what the trouble is							
34	After talking with the doctor, I know just how serious my illness is							
35	The doctor told me all I wanted to know about my illness							
36	I am not really certain about how to follow the doctors advice							
37	After talking with the doctor, I have a good idea of how long it will be before I am well again							
38	The doctor seemed interested in me as a person							
39	The doctor seemed warm and friendly to me							
40	The doctor seemed to take my problems seriously							
41	I felt embarrassed while talking with the doctor							
42	I felt free to talk to this doctor about private matters							
43	The doctor gave me a chance to say what was really on my mind							
44	I really felt understood by my doctor							
45	The doctor did not allow me to say everything I had wanted about my problems							
46	The doctor did not really understand my main reason for coming							
47	This is a doctor I would trust with my life							
48	The doctor seemed to know what (s)he was doing							
49	The doctor has relieved my worries about my illness							
50	The doctor seemed to know just what to do for my problem							
51	I expect that it will be easy for me to follow the doctors advice							
52	It may be difficult for me to do exactly what the doctor has told me to do							
53	I'm not sure the doctor's treatment will be worth the trouble it will take							

Thank you for taking the time to complete this questionnaire
